# Supplementary material for: A Likelihood Approach for Real-Time Calibration of Stochastic Compartmental Epidemic Models
Source: PLoS Comput Biol. 2017 Jan 17;13(1):e1005257. doi: 10.1371/journal.pcbi.1005257 (PMC5240920; doi:10.1371/journal.pcbi.1005257)
Supplement: S1 File — (TAR.GZ) [file pcbi.1005257.s014.tar.gz › HSPH_Online-SI-Revision/PF08/n1-mild/n1-mild_PF_table8.pdf]

1	R0	{1.27677, 7.89375}	{1.67516, 14.3876}	{{1.22218, 7.69796}, {2.38856, 19.9697}}	{33.237, 86.5609}	inf	4.	26.9495	{4.28034, 55.432}	585.926	0.742142	newly	2.	10.85	{2., 22.}	452.5	0.668576	Reff	0.856713	0.623628	{0.295615, 0.866271}	28.6513	ar	400.	755.59	{526., 1009.}	88.8975
2	R0	{1.33231, 10.5259}	{1.73223, 14.6042}	{{1.25362, 8.26949}, {2.35997, 19.9964}}	{32.7237, 50.1473}	inf	6.	23.5343	{3.05277, 48.1244}	312.694	0.511573	newly	4.	10.4	{2., 21.}	184.5	0.407266	Reff	0.714118	0.772944	{0.481559, 1.04894}	25.5171	ar	467.	731.93	{477., 978.}	58.3833
3	R0	{1.2273, 6.76944}	{1.64089, 12.6982}	{{1.10369, 6.25659}, {2.27582, 17.9732}}	{38.2938, 95.0372}	inf	9.	28.6584	{0.939891, 72.4077}	264.044	0.618907	newly	6.	12.51	{1., 27.}	159.167	0.550948	Reff	0.857883	0.671057	{0.354352, 0.941084}	28.8529	ar	314.	594.33	{249., 856.}	99.7548
4	R0	{1.41215, 17.5742}	{1.61144, 13.5106}	{{1.19494, 6.93268}, {2.20438, 19.8986}}	{21.3175, 27.6304}	inf	25.	21.9479	{0.561469, 56.0389}	76.1447	0.59413	newly	8.	9.37	{0., 21.}	85.875	0.508807	Reff	1.09159	0.634368	{0.350175, 0.835396}	41.886	ar	465.	731.92	{503., 974.}	58.0688
5	R0	{1.42222, 6.15886}	{1.66192, 12.8143}	{{1.12977, 6.05643}, {2.24606, 18.9248}}	{27.8853, 114.002}	inf	11.	15.9414	{0.422162, 38.1998}	109.421	0.540868	newly	5.	6.58	{0., 15.}	96.4	0.525788	Reff	0.742399	0.628127	{0.357673, 0.900875}	27.3418	ar	490.	603.33	{313., 853.}	43.6592
6	R0	{1.24363, 9.95596}	{1.69222, 15.2693}	{{1.23767, 8.04132}, {2.32056, 20.}}	{36.9222, 61.7176}	inf	11.	64.4113	{13.769, 120.607}	491.038	0.678009	newly	12.	25.81	{8., 42.}	128.583	0.33291	Reff	0.863079	0.802305	{0.447137, 1.08558}	23.4023	ar	411.	771.69	{543., 1007.}	87.7883
7	R0	{1.36478, 5.96056}	{1.65004, 12.5895}	{{1.13227, 5.71232}, {2.18932, 18.4113}}	{29.3411, 115.783}	inf	8.	25.4086	{0.672957, 58.6513}	268.286	0.646091	newly	5.	10.76	{0., 28.}	170.	0.57624	Reff	0.754723	0.641299	{0.339061, 0.853257}	24.3478	ar	484.	601.34	{278., 852.}	44.4174
8	R0	{1.36335, 17.6429}	{1.51588, 13.3117}	{{1.19532, 6.44816}, {1.96807, 19.7403}}	{18.4066, 28.343}	inf	28.	17.5003	{0.196528, 39.2649}	62.4471	0.718472	newly	12.	6.93	{0., 15.}	59.5833	0.727545	Reff	1.02251	0.659238	{0.402659, 0.8331}	35.5277	ar	484.	679.7	{493., 914.}	41.7603
9	R0	{1.26575, 5.28707}	{1.62139, 14.3157}	{{1.20732, 7.60665}, {2.14639, 20.}}	{30.0978, 170.768}	inf	6.	14.003	{0.864915, 34.7395}	184.556	0.529627	newly	5.	5.63	{0., 13.}	87.8	0.552725	Reff	0.669582	0.607711	{0.368608, 0.789719}	20.9822	ar	472.	739.02	{502., 984.}	57.0551
10	R0	{1.42942, 9.49905}	{1.69256, 14.7001}	{{1.23758, 7.715}, {2.39752, 20.}}	{26.2823, 63.6742}	inf	1.	17.8913	{0.61632, 40.0152}	1703.6	1.0591	newly	1.	7.	{1., 16.}	618.	0.737104	Reff	0.760451	0.591035	{0.298199, 0.806618}	25.9737	ar	468.	760.76	{521., 1002.}	62.8718
11	R0	{1.46827, 15.0983}	{1.54654, 13.472}	{{1.1831, 6.41782}, {2.03559, 19.9759}}	{18.5054, 28.808}	inf	22.	15.7745	{0.0597243, 36.5139}	70.1665	0.734616	newly	11.	6.46	{0., 15.}	64.7273	0.721322	Reff	1.12469	0.646969	{0.388373, 0.813084}	42.476	ar	495.	687.55	{481., 937.}	41.4848
12	R0	{1.25751, 13.279}	{1.58892, 13.7983}	{{1.20198, 6.99025}, {2.11635, 19.3751}}	{28.3303, 28.9358}	inf	16.	15.1497	{0.467079, 35.9956}	75.5541	0.597017	newly	9.	6.07	{0., 14.}	61.2222	0.635096	Reff	0.975828	0.622952	{0.379842, 0.814295}	36.1617	ar	366.	711.54	{503., 943.}	94.4098
13	R0	{1.3425, 9.16361}	{1.6545, 12.807}	{{1.11164, 5.62019}, {2.24606, 18.8228}}	{32.3211, 55.7139}	inf	2.	8.05553	{0.0881137, 21.1216}	356.784	0.685886	newly	0.1	3.22	{0., 7.}	3182.	1.04435	Reff	0.753143	0.58006	{0.326109, 0.792348}	26.769	ar	442.	602.71	{264., 855.}	52.509
14	R0	{1.18936, 6.20583}	{1.68058, 13.0719}	{{1.11164, 6.0993}, {2.42205, 18.9248}}	{44.5394, 116.416}	inf	3.	18.0117	{0.32395, 46.1605}	540.457	0.746737	newly	3.	7.41	{0., 16.}	202.333	0.624973	Reff	0.735024	0.613584	{0.317854, 0.817159}	27.1161	ar	382.	607.02	{279., 867.}	70.6859
15	R0	{1.27137, 9.78868}	{1.70116, 14.4645}	{{1.23379, 8.49668}, {2.34857, 20.}}	{35.2275, 55.1909}	inf	16.	37.1687	{7.70627, 81.9848}	156.445	0.40371	newly	13.	15.42	{3., 32.}	66.9231	0.329091	Reff	0.752651	0.682425	{0.385604, 0.922897}	22.9204	ar	496.	777.36	{555., 1007.}	57.8629
16	R0	{1.23102, 13.6452}	{1.63139, 14.0759}	{{1.21756, 6.88286}, {2.18164, 20.}}	{33.3101, 28.903}	inf	26.	22.6012	{0.316123, 49.5413}	67.9288	0.576087	newly	13.	8.87	{0., 21.}	56.2308	0.58249	Reff	0.853097	0.628714	{0.357406, 0.82225}	27.7872	ar	445.	739.94	{516., 986.}	66.8944
17	R0	{1.27213, 13.7452}	{1.66326, 14.3005}	{{1.22733, 7.24573}, {2.08844, 19.3211}}	{32.6004, 26.688}	inf	30.	36.2713	{3.42902, 77.7362}	71.6216	0.385065	newly	16.	15.53	{2., 31.}	54.4375	0.352372	Reff	0.957914	0.645625	{0.389516, 0.884612}	32.9544	ar	367.	757.12	{507., 956.}	106.3
18	R0	{1.25318, 11.2151}	{1.52831, 14.0208}	{{1.18378, 7.00515}, {1.93355, 20.}}	{24.0748, 42.6776}	inf	24.	17.2126	{0.434414, 39.7987}	68.3463	0.674974	newly	17.	7.18	{0., 17.}	63.0588	0.809236	Reff	0.833365	0.644671	{0.405721, 0.818813}	23.0389	ar	416.	694.78	{492., 912.}	67.0481
19	R0	{1.46006, 8.11496}	{1.71714, 13.3438}	{{1.11927, 6.25107}, {2.27287, 19.0665}}	{29.1051, 74.5107}	inf	20.	19.5176	{0.7454, 49.0582}	76.4731	0.528508	newly	12.	7.74	{0., 18.}	61.3333	0.581112	Reff	0.762151	0.609345	{0.340523, 0.849895}	30.1943	ar	496.	619.24	{250., 864.}	46.5242
20	R0	{1.14273, 6.71261}	{1.61905, 14.835}	{{1.19474, 7.72471}, {2.37775, 20.}}	{41.7823, 122.281}	inf	15.	25.9423	{3.58423, 53.0074}	119.492	0.426659	newly	11.	10.39	{1., 20.}	60.2727	0.409466	Reff	0.742775	0.644456	{0.301135, 0.897428}	23.5262	ar	362.	722.73	{480., 980.}	99.6492
21	R0	{1.42432, 8.23905}	{1.70797, 14.1772}	{{1.22423, 8.33131}, {2.36694, 19.763}}	{25.9241, 76.7127}	inf	6.	25.5641	{3.10667, 56.4427}	343.993	0.518859	newly	5.	11.2	{2., 25.}	152.	0.364434	Reff	0.853168	0.797031	{0.500246, 1.11289}	22.6101	ar	405.	737.78	{536., 947.}	82.2815
22	R0	{1.29386, 17.863}	{1.63932, 14.2791}	{{1.19762, 7.92286}, {2.32062, 20.}}	{29.8413, 23.9761}	inf	35.	31.0982	{2.44848, 64.9441}	62.8596	0.441683	newly	16.	12.68	{2., 29.}	57.75	0.450103	Reff	0.98204	0.656571	{0.33397, 0.880737}	33.1961	ar	488.	736.92	{504., 987.}	52.9467
23	R0	{1.39274, 13.0167}	{1.57948, 14.5129}	{{1.20865, 8.15538}, {2.10383, 20.}}	{20.7443, 30.9314}	inf	19.	18.4502	{1.34989, 41.4691}	73.3832	0.495265	newly	8.	7.22	{0., 16.}	69.75	0.516381	Reff	0.799433	0.632036	{0.376225, 0.82699}	23.1702	ar	475.	728.6	{499., 962.}	54.0379
24	R0	{1.32178, 15.8991}	{1.59264, 14.1735}	{{1.21809, 7.3731}, {2.17653, 19.6498}}	{24.7958, 23.3497}	inf	28.	24.5431	{1.01686, 53.1317}	69.5866	0.545448	newly	13.	9.71	{0., 21.}	59.9231	0.560019	Reff	0.810251	0.643843	{0.346863, 0.839821}	23.5676	ar	471.	716.62	{486., 961.}	53.4268
25	R0	{1.37568, 17.8427}	{1.59372, 13.5074}	{{1.1847, 7.13353}, {2.36421, 19.4044}}	{23.6878, 27.4685}	inf	24.	11.1411	{0.467556, 29.0879}	66.8281	0.76732	newly	8.	4.87	{0., 13.}	69.125	0.726574	Reff	1.00837	0.615146	{0.293408, 0.79627}	38.9962	ar	398.	711.74	{492., 979.}	78.9648
26	R0	{1.20993, 6.4656}	{1.64433, 15.1664}	{{1.19969, 7.77549}, {2.38691, 20.}}	{36.678, 136.698}	inf	2.	23.2123	{1.40656, 51.8138}	1073.22	0.916465	newly	1.	8.9	{1., 20.}	804.	0.841095	Reff	0.803394	0.625916	{0.32006, 0.846362}	24.8759	ar	345.	738.3	{504., 996.}	114.
27	R0	{1.20548, 13.8003}	{1.63362, 14.2644}	{{1.2012, 7.04343}, {2.37105, 20.}}	{36.2584, 29.4407}	inf	23.	14.942	{0.174226, 36.5107}	69.6575	0.717421	newly	10.	6.06	{0., 15.}	64.	0.730036	Reff	0.817315	0.609945	{0.324674, 0.808095}	26.1089	ar	350.	718.22	{483., 984.}	105.206
28	R0	{1.3176, 14.6594}	{1.63555, 13.5254}	{{1.20709, 6.98567}, {2.13924, 19.6475}}	{27.6221, 27.76}	inf	16.	31.4093	{0.528364, 71.4576}	150.534	0.580483	newly	8.	12.8	{1., 28.}	113.	0.513891	Reff	1.06989	0.665099	{0.346266, 0.891644}	37.8349	ar	362.	726.58	{503., 963.}	100.713
29	R0	{1.28202, 6.23047}	{1.58123, 14.5395}	{{1.21517, 7.87835}, {2.16222, 20.}}	{25.5625, 133.361}	inf	4.	22.762	{2.09117, 47.2975}	488.563	0.667055	newly	5.	9.07	{1., 19.}	124.6	0.448183	Reff	0.782032	0.649472	{0.361307, 0.850783}	21.3295	ar	391.	715.42	{504., 951.}	82.9719
30	R0	{1.28177, 8.83252}	{1.64724, 12.3837}	{{1.11164, 5.3704}, {2.29887, 18.765}}	{35.2543, 58.2461}	inf	18.	18.9955	{0.210224, 45.509}	91.4136	0.678444	newly	11.	7.79	{0., 18.}	71.7273	0.702312	Reff	0.76778	0.641143	{0.357996, 0.839425}	27.6355	ar	427.	591.17	{257., 868.}	54.6066
31	R0	{1.35886, 5.97708}	{1.79289, 14.8448}	{{1.23365, 8.07649}, {2.42215, 20.}}	{35.2059, 149.578}	inf	0.1	34.5766	{6.0268, 78.538}	34 476.6	2.36784	newly	0.1	14.08	{4., 30.}	13 982.	2.00759	Reff	0.69166	0.72382	{0.43638, 1.07474}	28.2261	ar	491.	799.92	{557., 1001.}	63.6334
32	R0	{1.27601, 17.9804}	{1.63137, 14.0543}	{{1.20622, 6.99298}, {2.11139, 20.}}	{30.0632, 26.1658}	inf	36.	21.1661	{0.269912, 50.6741}	62.7903	0.730149	newly	15.	8.13	{0., 19.}	59.8	0.70308	Reff	0.962112	0.632078	{0.371143, 0.835166}	34.3031	ar	445.	729.79	{505., 965.}	64.1506
33	R0	{1.3368, 11.3235}	{1.67743, 14.152}	{{1.21904, 7.60254}, {2.30392, 20.}}	{29.2499, 41.3811}	inf	13.	25.3061	{0.82747, 51.4075}	145.715	0.536523	newly	7.	10.04	{1., 20.}	96.2857	0.471483	Reff	0.788712	0.609601	{0.315257, 0.828045}	25.6822	ar	417.	757.85	{505., 985.}	81.94
34	R0	{1.21021, 11.3635}	{1.62622, 14.3983}	{{1.19053, 8.1442}, {2.23824, 20.}}	{35.1994, 39.2841}	inf	18.	22.5403	{0.919746, 47.6302}	88.1601	0.488111	newly	15.	8.79	{0., 20.}	61.2667	0.615651	Reff	0.905237	0.642156	{0.388675, 0.842043}	29.4153	ar	327.	736.8	{513., 957.}	125.321
35	R0	{1.53382, 10.8866}	{1.6404, 14.7341}	{{1.21119, 7.84968}, {2.19071, 20.}}	{21.3352, 46.6397}	inf	21.	28.6042	{3.6348, 55.4709}	82.9201	0.401805	newly	15.	11.27	{1., 21.}	49.1333	0.426668	Reff	0.852804	0.628497	{0.369942, 0.841739}	27.3445	ar	476.	746.17	{514., 988.}	57.8592
36	R0	{1.20423, 6.83844}	{1.74931, 13.3211}	{{1.14292, 6.31117}, {2.43839, 19.0531}}	{47.3326, 99.8678}	inf	0.1	18.3305	{0.653065, 45.0094}	18 231.7	1.91705	newly	0.1	7.27	{0., 16.}	7196.	1.51939	Reff	0.70327	0.581544	{0.320077, 0.830868}	27.5164	ar	416.	637.75	{327., 880.}	64.0986
37	R0	{1.43629, 10.1548}	{1.51973, 14.1001}	{{1.18965, 7.98352}, {1.94374, 20.}}	{15.9481, 50.0687}	inf	12.	24.278	{2.93545, 52.0181}	144.442	0.479862	newly	8.	9.9	{1., 20.}	81.	0.42372	Reff	0.963751	0.673853	{0.423974, 0.848231}	30.0801	ar	367.	703.76	{519., 917.}	91.7602
38	R0	{1.37504, 8.93428}	{1.73243, 13.2195}	{{1.14292, 5.40945}, {2.43839, 19.0531}}	{33.0029, 62.931}	inf	7.	21.7892	{0.503331, 58.6813}	258.817	0.634319	newly	5.	8.56	{0., 23.}	132.	0.568276	Reff	0.717771	0.598932	{0.329207, 0.839425}	27.1751	ar	483.	634.54	{329., 872.}	47.9048
39	R0	{1.51637, 9.36742}	{1.6591, 12.8546}	{{1.11228, 5.40945}, {2.27582, 18.9248}}	{25.5971, 54.743}	inf	9.	15.2848	{0.198575, 36.0666}	142.313	0.646495	newly	4.	6.03	{0., 13.}	122.75	0.620757	Reff	0.884044	0.598056	{0.328488, 0.811422}	33.7414	ar	441.	608.65	{323., 858.}	52.678
40	R0	{1.23658, 9.82869}	{1.65491, 12.5932}	{{1.12977, 5.62019}, {2.23964, 18.765}}	{37.6977, 49.7428}	inf	1.	21.4564	{0.434797, 51.9853}	2064.13	1.0684	newly	1.	8.8	{0., 21.}	808.	0.842776	Reff	0.724636	0.635431	{0.357828, 0.848803}	24.5517	ar	414.	593.65	{255., 853.}	58.9879
41	R0	{1.24247, 5.4242}	{1.71585, 12.7614}	{{1.12882, 6.55128}, {2.32014, 18.4377}}	{42.182, 137.18}	inf	5.	27.8853	{2.12023, 56.5873}	483.276	0.660796	newly	5.	12.16	{1., 28.}	182.4	0.476603	Reff	0.821273	0.622752	{0.343097, 0.859507}	30.4609	ar	345.	624.73	{291., 872.}	89.8232
42	R0	{1.28208, 4.40223}	{1.67916, 13.6464}	{{1.06468, 6.71734}, {2.22731, 18.9003}}	{36.6797, 210.07}	inf	0.1	22.6754	{0.773748, 51.992}	22 577.6	2.01985	newly	0.1	9.08	{0., 22.}	9002.	1.63378	Reff	0.847455	0.620174	{0.333012, 0.8601}	31.0238	ar	339.	612.25	{255., 853.}	92.1268
43	R0	{1.56773, 6.50474}	{1.74586, 12.151}	{{1.10693, 6.0993}, {2.46976, 17.5057}}	{26.2756, 94.4058}	inf	3.	18.5208	{1.17504, 44.3716}	542.543	0.698192	newly	2.	7.55	{1., 17.}	299.5	0.567668	Reff	0.790136	0.583499	{0.301938, 0.904726}	32.8815	ar	498.	632.43	{219., 889.}	47.9739
44	R0	{1.10489, 6.01941}	{1.673, 15.0423}	{{1.20808, 8.12413}, {2.39113, 20.}}	{51.4177, 149.897}	inf	1.	18.7567	{1.95424, 43.164}	1780.45	1.05775	newly	0.1	7.33	{1., 16.}	7244.	1.61313	Reff	0.724808	0.591312	{0.294611, 0.798273}	24.105	ar	346.	746.89	{515., 991.}	115.864
45	R0	{1.25888, 14.1926}	{1.62716, 14.6714}	{{1.21668, 7.7578}, {2.35376, 20.}}	{30.9132, 26.5838}	inf	17.	19.7768	{0.715323, 39.9531}	90.5664	0.538483	newly	7.	7.59	{0., 18.}	80.7143	0.544293	Reff	0.682313	0.619842	{0.326031, 0.820657}	21.0415	ar	471.	740.26	{526., 997.}	57.5372
46	R0	{1.45292, 16.616}	{1.63959, 14.9583}	{{1.21034, 8.33176}, {2.26003, 19.7755}}	{22.7148, 19.93}	inf	28.	39.0814	{8.38919, 74.6675}	78.781	0.336155	newly	9.	16.21	{3., 31.}	107.889	0.373563	Reff	1.03012	0.664074	{0.348681, 0.920212}	35.5733	ar	494.	754.63	{540., 1001.}	53.8117
47	R0	{1.22528, 16.6033}	{1.47273, 14.3798}	{{1.18873, 7.12632}, {1.90309, 20.}}	{21.6584, 25.376}	inf	30.	32.5953	{1.74809, 69.6242}	70.144	0.470744	newly	10.	12.57	{1., 28.}	79.3	0.442853	Reff	1.00963	0.721529	{0.45727, 0.8889}	28.6035	ar	368.	669.66	{470., 896.}	81.9728
48	R0	{1.1669, 6.92715}	{1.62636, 14.2535}	{{1.23372, 8.05012}, {2.16082, 20.}}	{39.4884, 107.722}	inf	6.	17.7117	{0.418625, 39.8139}	248.496	0.616501	newly	3.	6.89	{0., 19.}	185.667	0.616801	Reff	0.735147	0.615993	{0.352783, 0.790183}	20.4148	ar	416.	741.21	{524., 965.}	78.1755
49	R0	{1.32749, 16.0887}	{1.56189, 14.3097}	{{1.1875, 6.98109}, {2.10515, 20.}}	{22.9539, 25.6115}	inf	18.	26.8035	{1.08845, 57.1798}	109.925	0.542425	newly	3.	10.17	{0., 23.}	277.	0.633455	Reff	1.06863	0.678292	{0.374275, 0.893603}	36.5269	ar	353.	692.89	{456., 945.}	96.2861
50	R0	{1.29636, 15.2609}	{1.62005, 14.959}	{{1.19927, 9.28428}, {2.29582, 20.}}	{27.9998, 22.6942}	inf	33.	19.7808	{1.14259, 43.7736}	56.6883	0.581214	newly	13.	8.06	{0., 17.}	57.2308	0.627109	Reff	0.735036	0.611015	{0.333892, 0.817776}	22.263	ar	470.	738.38	{529., 976.}	57.5915
